# Increased expression of ficolin-1 is associated with airway obstruction in asthma

**DOI:** 10.1186/s12890-023-02772-2

**Published:** 2023-11-24

**Authors:** Pengfei Gao, Kun Tang, Yanjiao Lu, Meijia Wang, Wei Wang, Tongsheng Wang, Yuxia Sun, Jianping Zhao, Yimin Mao

**Affiliations:** 1 Department of Respiratory and Critical Care Medicine, The First Affiliated Hospital, Henan University of Science and Technology, Luoyang, Henan China; 2 Department of Respiratory and Critical Care Medicine, Tongji Hospital of Tongji Medical College, Huazhong University of Science and Technology, Wuhan, Hubei China; 3 Department of Pulmonary and Critical Care Medicine, The First Affiliated Hospital of Sun Yat-Sen University, Guangzhou, China

**Keywords:** Asthma, Ficolins, Pulmonary function, Asthma control questionnaire

## Abstract

**Background:**

The activated complement cascade is involved in asthmatic airway inflammation. Ficolins are essential for innate immunity and can activate the complement lectin pathway. Despite this, the significance of ficolins in asthma has yet to be determined. This study aimed to explore the presence of ficolins in individuals with asthma and to determine the relationship between ficolins and clinical characteristics.

**Methods:**

For the study, 68 asthmatic patients and 30 healthy control subjects were recruited. Enzyme-linked immunosorbent assay was used to determine plasma ficolin-1, ficolin-2, and ficolin-3 concentrations both before and after inhaled corticosteroid (ICS) therapy. Further, the associations of plasma ficolin-1 level with pulmonary function and asthma control questionnaire (ACQ) score were examined in the asthma patients.

**Results:**

Patients with asthma exhibited significantly elevated plasma ficolin-1 levels (median, 493.9 ng/mL; IQR, 330.2–717.8 ng/mL) in comparison to healthy controls (median, 330.6 ng/mL; IQR, 233.8–371.1 ng/mL). After ICS treatment, plasma ficolin-1 (median, 518.1 ng/mL; IQR, 330.2–727.0 ng/mL) in asthmatic patients was significantly reduced (median, 374.7 ng/mL; IQR, 254.8–562.5 ng/mL). Additionally, ficolin-1 expressions in plasma were significantly correlated with pulmonary function parameters and ACQ score in asthmatic patients. Asthma patients with higher plasma ficolin-1 levels demonstrated poorer lung function than those with lower plasma ficolin-1 levels.

**Conclusions:**

The results revealed that asthmatic patients had higher plasma ficolin-1 concentrations, which decreased after ICS treatment and were linked to their lung function, implying a potential involvement of ficolin-1 in asthma pathogenesis.

## Introduction

Asthma is a widespread and heterogeneous disorder characterized by persistent airway inflammation [1]. Recent studies have indicated that the prevalence of asthma has been on the rise for many years and currently affects 45.7 million adults in China [2]. The importance of innate immune pathways in controlling adaptive immune responses has brought to light the role of innate immune pathways in the pathogenesis of asthma [3], with the complement system acting as a bridge between the two [4]. In recent years, the involvement of complement activation in allergic airway inflammation has been widely acknowledged [5]. Additionally, high levels of plasma complement C3 have been linked to an increased risk of asthma hospitalizations and exacerbations in asthmatic patients, suggesting a causal role of the complement system in the development of asthma [6].

Ficolins are components of the innate immune system, which activate one of the three pathways of complement activation, known as the lectin pathway [7]. Three human ficolins have been identified: ficolin-1 (M-ficolin), ficolin-2 (L-ficolin), and ficolin-3 (H-ficolin) [8], as well as two murine variants, ficolin A and B [9]. According to phylogenetic analysis, ficolin B is the homologue of human ficolin-1, and ficolin A is closely related to human ficolin-2 [10]. The gene encoding the mouse ficolin-3 is a pseudogene [11]. Structurally, ficolin genes consist of a basic homotrimer, where each chain comprises an N-terminal region, followed by a collagen-like domain and a C-terminal fibrinogen-like domain [12]. The collagen-like domain interacts with the MBL-associated serine proteases (MASPs), and the ficolin-MASP complex binds to carbohydrates present on the surface of microorganisms to initiate complement activation through the lectin pathway [13]. Ficolin-1 is mainly produced in peripheral leukocytes, the spleen, and the lung [14], while ficolin-2 is expressed solely by hepatocytes in the liver and secreted into the bloodstream, and ficolin-3 is synthesized by hepatocytes in the liver and the lung, and can also be found in the serum [15].

As soluble pattern recognition receptors, ficolins have been observed to bind to various clinically relevant microorganisms, such as fungi, bacteria, and viruses, suggesting that they may play a role in defending the host against infection. However, the same components of the humoral innate immune response can be pathogenic in autoimmune and chronic inflammatory disorders [16]. The accumulated evidence, including mouse models and clinical studies, suggests that genetic variation or insufficient expression of ficolins is associated with increased susceptibility to various infections, such as *Aspergillus fumigatus* [17], *Mycobacterium avium* [18], *Mycobacteria tuberculosis* [19], and *Streptococcus pneumoniae* [20]. Moreover, a growing body of research has indicated that ficolins play a significant role in the onset and progression of several autoimmune diseases, including systemic lupus erythematosus (SLE), rheumatoid arthritis, systemic sclerosis, type 1 diabetes, and inflammatory bowel disease, as reviewed by Wang et al. [21]. However, no investigations have been conducted to examine the involvement of ficolins in asthma.

Taken together, it is tempting to speculate that ficolins may be involved in the pathogenesis of asthma, and associated with the clinical features. In this research, we investigated the expression of ficolins in asthmatic patients and found that only ficolin-1 plasma levels were substantially raised in asthmatic patients compared to healthy individuals. Additionally, the correlation of ficolin-1 expressions with pulmonary function and clinical syndromes in asthma patients was also analyzed.

## Methods

### Subjects

In the current case–control study, 30 healthy individuals and 68 asthma patients were recruited from Tongji Hospital (Wuhan, China) for the study, as previously described [22], with the following inclusion and exclusion criteria. All participants provided written informed consent, and the study was approved by the ethics committee of Tongji Hospital, Huazhong University of Science and Technology (IRB ID: 20,150,503). Healthy individuals had no prior history of respiratory illness or bronchial hyper-responsiveness. Asthmatic patients were diagnosed according to Global Initiative for Asthma guidelines, with a physician-confirmed diagnosis and evidence of airway hyper-responsiveness (provacative dose of methacholine < 2.5 mg to lower forced expired volume in 1 s (FEV1) by 20%) and/or reversibility in FEV1% predicted > 12% after inhalation of 200 μg of salbutamol. Spirometry was applied for lung function test using the same spirometer (Jaeger Co., Wurzburg, Germany), in accordance with the guidelines recommended in the American Thoracic Society (ATS)/European Respiratory Society (ERS) Standardization [23]. Patients with any other lung, heart, kidney, liver, or collagen diseases were excluded, as were smokers or ex-smokers with a history > 10 pack years. No patient was undergoing a course of oral or inhaled corticosteroid (ICS), or had any respiratory infection symptoms in the four weeks before the study. Demographic information, pulmonary function tests, and plasma samples were collected for ficolins measurement at baseline, with blood samples taken from the asthmatic patients after four weeks of inhaled corticosteroid (ICS) treatment (budesonide, 160 μg twice a day). Plasma samples were processed at 2000 g for 15 min at four °C and, stored at − 80 °C before analysis and thawed only once prior to use.

### Measurement of plasma ficolin levels

Plasma ficolin-1, ficolin-2, and ficolin-3 were measured using an Enzyme-linked immunosorbent assay (ELISA) per the manufacturer's instructions (Hycult Biotech, Uden, Netherlands). Prior to measurement, the plasma samples were diluted in a ratio of 1:20 for ficolin-1 and ficolin-2 and 1:200 for ficolin-3. The standard curves for these assays ranged from 3.1 to 200 ng/mL for ficolin-1, 15.6 to 1000 ng/mL for ficolin-2, and 7.8 to 500 ng/mL for ficolin-3, respectively.

### Determination of fractional exhaled nitric oxide (FeNO)

According to the American Thoracic Society's guidelines [24], the IOX MINO (Aerocrine AB, Sweden) analytical device was utilized to measure FeNO concentration. Patients were instructed to inhale air without NO until reaching total vital capacity, then exhale it continuously at 50 ml/s to the instrument.

### Asthma Control Questionnaire (ACQ)

The ACQ questionnaire comprises seven items that assess the typical asthma symptoms experienced in the past week, including nocturnal awakening, severity of symptoms, activity limitation, frequency of dyspnea and wheezing, use of rescue medications, and lung function parameters [25]. Each item is rated on a 7-point scale, from 0 (totally controlled) to 6 (extremely poorly controlled). The ACQ score, used to measure the degree of asthma control, is obtained by adding all scores and dividing by the number of items.

### Statistical analysis

As previously described [22], unpaired t-tests were utilized to analyze normally distributed data, with mean and standard deviation results. For non-normally distributed data, results were expressed as a median and interquartile range, and Kruskal–Wallis and Mann–Whitney nonparametric tests were employed to compare across groups. Furthermore, Fisher exact tests were conducted for categorical data, and Spearman Rank Order Correlation was used to assess variable correlations. Receiver operating characteristic (ROC) curve analysis was performed, and areas under the curves (AUCs) were calculated to evaluate the diagnostic efficiency of ficolin-1 levels. All statistical analyses were performed using GraphPad Prism 6 and SPSS Statistical Software (version 22.0). Statistical significance was determined at a *p*-value < 0.05.

## Results

### Subject characteristics

The present study recruited 68 asthma patients and 30 healthy controls (Table 1). There was no significant disparity between subjects with asthma and heathy controls regarding gender, age, and body mass index (BMI). As anticipated, the asthma patients had poorer lung function than the healthy controls.

**Table 1:** Overall subject characteristics

Characteristics	Healthy Control Subjects	Subjects with Asthma	*P* value
Number	30	68	NA
Sex (F/M)	17/13	33/35	0.515
Age (y)	42.93 ± 16.78	41.62 ± 11.19	0.6486
BMI (kg/m^2^)	23.61 ± 3.40	23.15 ± 3.74	0.2883
Lung function			
FEV_1_ (L)	3.36 ± 0.67	2.57 ± 0.87	< 0.0001
FEV_1_% predicted	104.30 ± 10.37	84.50 ± 21.57	< 0.0001
FVC (L)	4.09 ± 0.82	3.74 ± 1.00	0.0806
FVC % predicted	105.80 ± 10.56	103.40 ± 17.22	0.4088
FEV1/FVC (%)	82.83 ± 5.82	67.97 ± 12.49	< 0.0001

Values are presented as mean ± SD or median (interquartile range). *NA* Not applicable

### Increased ficolin-1 expression in asthma

**Fig. 1:**
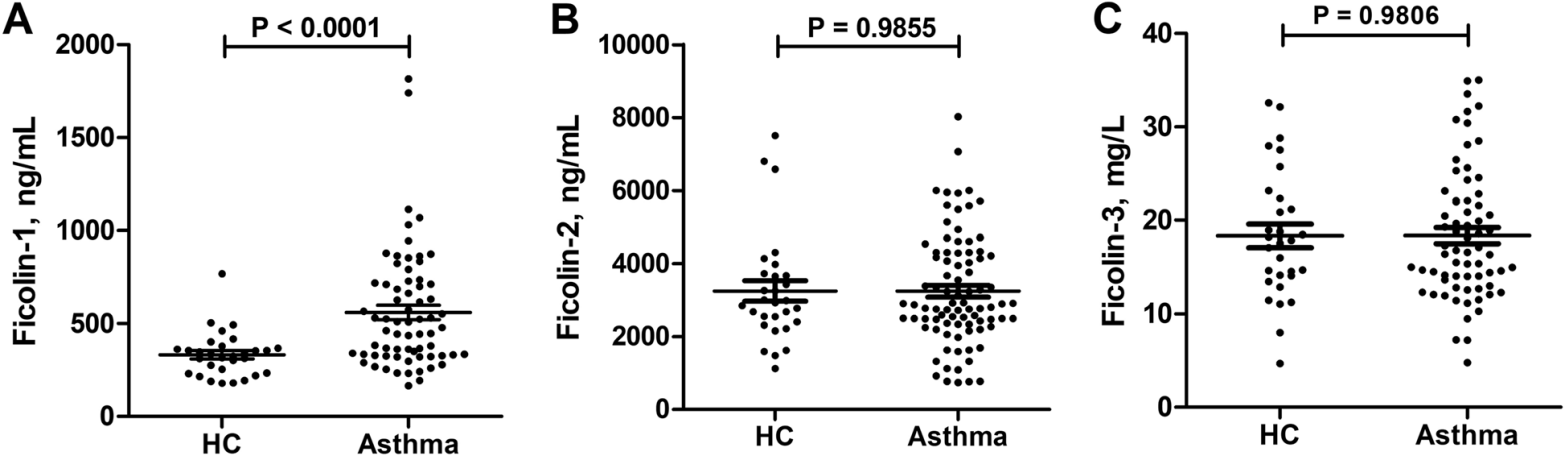
Plasma ficolin levels in healthy controls and asthmatic patients. Data are presented as median (interquartile range). Plasma ficolin-1 (**A**), ficolin-2 (**B**), and ficolin-3 (**C**) levels. *HC* Healthy control

Plasma ficolin-1 concentrations in the asthma group (median, 493.9 ng/mL; IQR, 330.2–717.8 ng/mL) were significantly higher than those of the healthy control group (median, 330.6 ng/mL; IQR, 233.8–371.1 ng/mL) (Fig. 1A). In contrast, there was no significant difference in ficolin-2 and ficolin-3 plasma levels between the asthma and control groups (Fig. 1B and C).

### The responsiveness of ficolins to ICS treatment

**Fig. 2:**
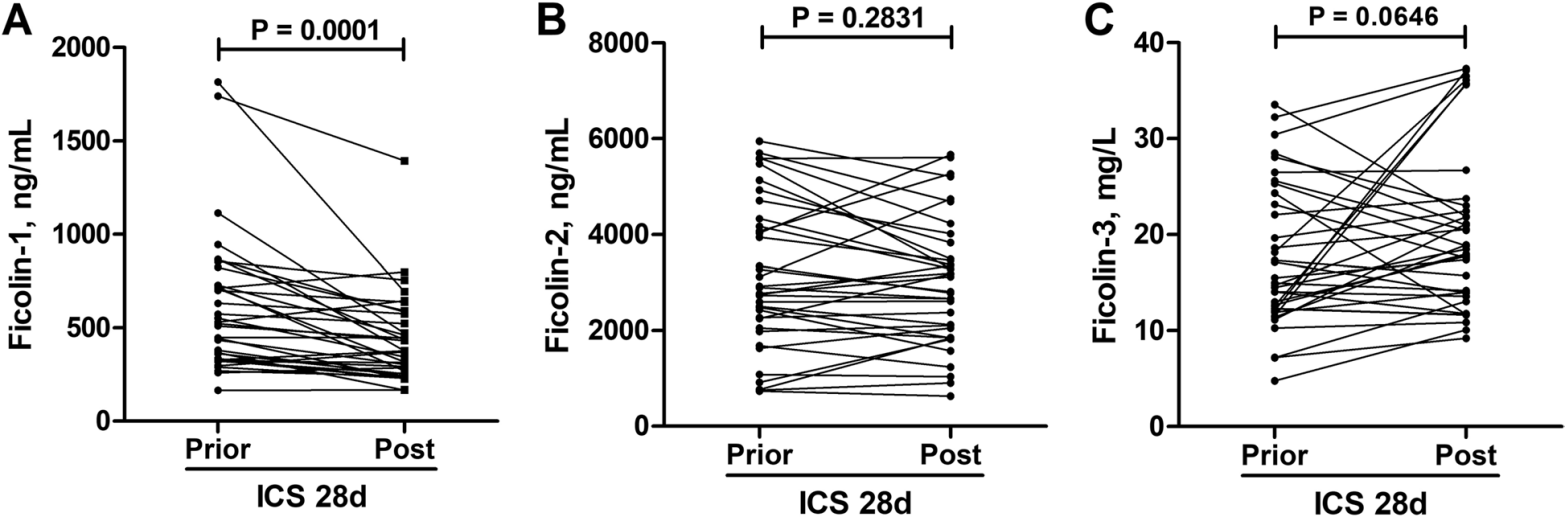
Plasma ficolin levels before and after ICS treatment for 4 weeks in asthmatic patients. Plasma ficolin-1 (**A**), ficolin-2 (**B**), and ficolin-3 (**C**) levels. *ICS* Inhaled corticosteroid

Thirty-six patients were followed up, and their peripheral blood was collected again after four weeks. We observed a significant decrease in the plasma ficolin-1 levels (median, 518.1 ng/mL; IQR, 330.2–727.0 ng/mL) of asthmatic patients after ICS treatment (median, 374.7 ng/mL; IQR, 254.8–562.5 ng/mL) (Fig. 2A). However, there was no significant alteration in the plasma ficolin-2 and ficolin-3 concentrations of asthmatic patients before and after inhaled corticosteroid ICS treatment (Fig. 2B and C).

### Correlations between plasma ficolin-1 and pulmonary function in patients with asthma

**Fig. 3:**
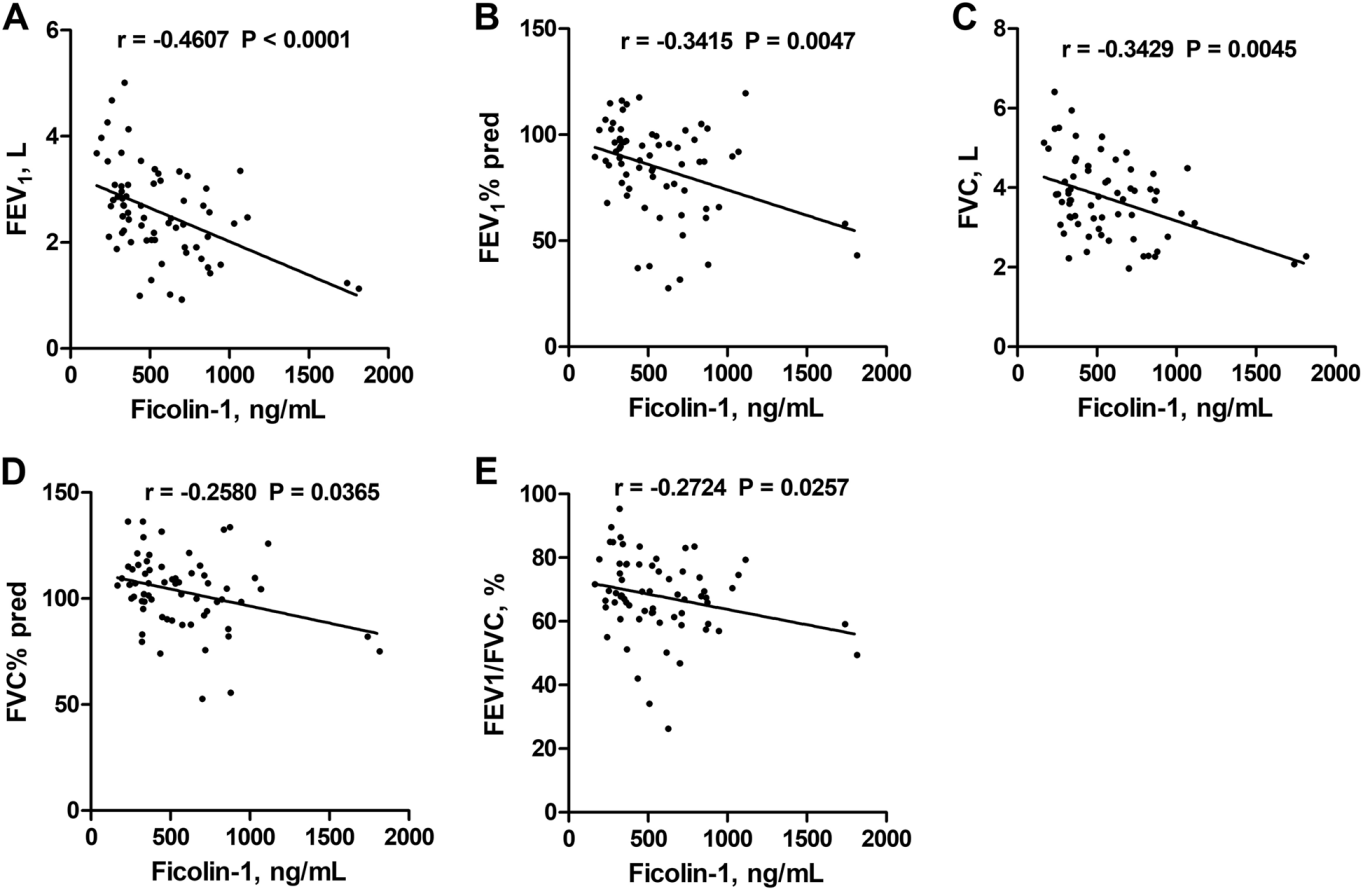
Correlations between plasma ficolin-1 and pulmonary function in patients with asthma. Correlation between plasma ficolin-1 and FEV_1_ (**A**), FEV_1_% pred (**B**), FVC (**C**), FVC% pred (**D**), and FEV_1_/ FVC (**E**). *FEV*_*1*_  forced expired volume in 1 s, *FVC* Forced vital capacity

**Fig. 4:**
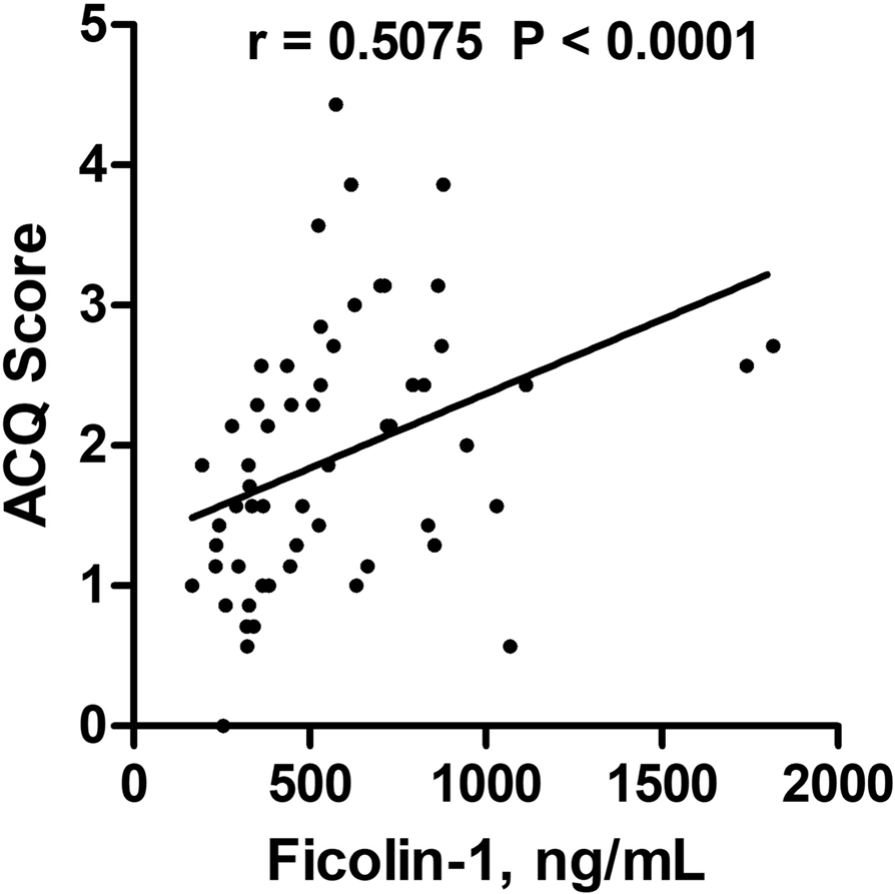
Correlation between plasma ficolin-1 and ACQ in patients with asthma. *ACQ* Asthma control questionnaire

To gain a deeper understanding of the link between ficolin-1 and clinical indices, we examined the correlation between plasma ficolin-1 levels and pulmonary function. Our study revealed that plasma ficolin-1 levels were inversely correlated with forced expired volume in 1 s (FEV1) (*r* = -0.4607, *P* < 0.0001), FEV1 to total predicted value ratio (FEV1% pred) (*r* = -0.3415, *P* = 0.0047), forced vital capacity (FVC) (*r* = -0.3429, *P* = 0.0045), FVC to total predicted value ratio (FVC% pred) (*r* = -0.2580, *P* = 0.0365), and FEV1/ FVC (*r* = -0.2724, *P* = 0.0257) (Fig. 3A-E). This suggests that asthmatic patients with higher ficolin-1 expression had poorer lung function. Additionally, ficolin-1 expression was positively correlated with ACQ score (*r* = 0.5075, *P* < 0.0001) (Fig. 4).

### Comparison of clinical characteristics in asthma patients with low and high plasma ficolin-1 levels

We classified asthma patients according to their plasma ficolin-1 levels, using the lower limit of the upper quartile for healthy control subjects (371.1 ng/mL) as a cut-point. We found that those with high ficolin-1 levels were significantly older (*P* = 0.0075) and had a higher BMI (*P* = 0.0401) than those with low levels. Additionally, asthma patients with high ficolin-1 levels had worse lung function than those with low plasma ficolin-1 levels. However, there were no significant differences in total IgE and FeNO. Though there was a tendency for patients with high plasma ficolin-1 levels to have higher levels of blood neutrophils and eosinophils, this did not reach statistical significance (Table 2).

**Table 2:** Asthmatic patient characteristics by Ficolin-1 expression

Characteristics	Low Ficolin-1	High Ficolin-1	*P* value
Number	26	42	NA
Sex (F/M)	12/14	21/21	0.8067
Age (y)	37.08 ± 10.81	44.43 ± 10.59	0.0075
BMI (kg/m^2^)	21.94 ± 3.50	23.87 ± 3.72	0.0401
Lung Function			
FEV_1_ (L)	3.09 ± 0.80	2.24 ± 0.74	< 0.0001
FEV_1_% predicted	95.31 ± 12.70	77.65 ± 23.30	0.0007
FVC (L)	4.18 ± 1.02	3.47 ± 0.89	0.0037
FVC % predicted	109.10 ± 13.85	99.92 ± 18.28	0.0351
FEV1/FVC (%)	73.01 ± 10.58	64.77 ± 12.68	0.0075
Methacholine PD20 (mg)	0.14 (0.01–1.39)	0.12 (0.03–0.67)	0.8297
Total IgE (IU/ml)	88.60 (39.88–298.80)	120.30 (49.40–255.00)	0.7961
FeNO (ppb)	71.00 (24.00–123.00)	53.00 (31.00–100.50)	0.3874
Blood neutrophils (10^9^/L)	3.53 ± 1.47	4.24 ± 1.63	0.0902
Blood eosinophils (10^9^/L)	0.23 (0.05–0.37)	0.30 (0.13–0.51)	0.0634

Values are presented as mean ± SD or median (interquartile range). *NA* Not applicable

### Diagnostic value of plasma ficolin-1 for asthma

**Fig. 5:**
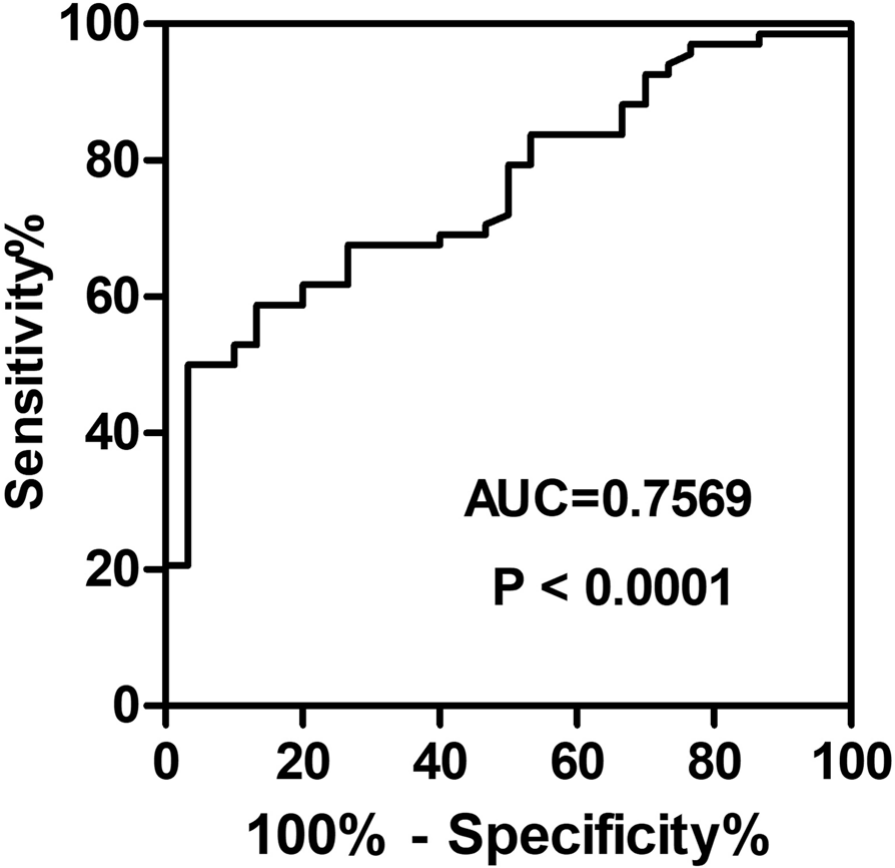
Diagnostic value of plasma ficolin-1 for asthma. ROC curve for the sensitivity and specificity evaluation of plasma ficolin-1 to diagnose asthma. *ROC*  Receiver operating characteristic

We conducted further analysis to assess the diagnostic value of ficolin-1 in asthma using the receiver operating characteristic (ROC) curve. The area under curve (AUC) value was 0.76 [95% confidence interval (CI): 0.66–0.85], and the optimal cut-off value was 506.7 ng/mL. This cut-off level resulted in a sensitivity and specificity of 50% and 96.67%, respectively (Fig. 5). The ROC curve of plasma ficolin-1 revealed a satisfactory diagnostic capacity in asthma.

## Discussion

Our data was the initial evidence of ficolin-1 expression in asthma patients, with asthmatic patients displaying significantly higher plasma ficolin-1 levels than healthy controls. Furthermore, plasma ficolin-1 levels in asthmatic patients were significantly reduced after ICS treatment. Additionally, there were significant associations between plasma ficolin-1 levels and clinical indices, including lung function and ACQ score, with those with high plasma ficolin-1 levels having poor lung function.

It has been well established that uncontrolled activation of the complement system in the airways is a contributing factor to asthma pathogenesis [5, 26, 27]. Ficolins are pattern recognition molecules with collagen-like and fibrinogen-like domains and can activate the complement system through the lectin pathway [28]. However, no data on this topic has been available on asthma patients. Another trigger of the lectin pathway, mannose binding lectin (MBL), has been studied about allergic inflammation, though the results have been somewhat contradictory. Allergen extracts have been found to bind purified MBL and activate the complement cascade in vitro [29]. In contrast, MBL deficiency has been found to reduce airway hyperresponsiveness, inflammation, and type 2 cytokine levels in a model of chronic fungal asthma [30], suggesting that the MBL-induced lectin pathway is involved in allergic disorders. Uguz et al. reported that serum MBL levels were significantly higher in children with asthma and correlated with peripheral blood eosinophils in these patients [31]. Furthermore, MBL levels have been found to differ in asthmatic children of varying severity [32]. Nagy et al. concluded that MBL2 gene polymorphisms are significant in the susceptibility to asthma in children infected with C. *pneumoniae* [33]. However, MBL2 gene polymorphisms have been reported not to be associated with the atopy status and asthma phenotype in adults [34, 35].

It is possible to hypothesize that ficolins, which activate the complement lectin pathway, may be involved in the development of asthma. A prospective study found that serum ficolin-2 concentrations were significantly lower in patients with allergic airway disease and respiratory infections. However, the gap between those with allergic disorders and healthy controls appeared less pronounced [36]. The authors proposed that decreased ficolin-2 levels could make individuals more vulnerable to respiratory infections, which could result in allergic diseases [36]. Studies examining the direct involvement of ficolins in allergic inflammation are limited. To address this, we conducted a study to evaluate the expression of all three ficolins in asthma. Ficolin-2 and ficolin-3 are mainly synthesized in the liver, while ficolin-1 is the only human ficolin produced in the bone marrow and expressed primarily to granulocytes, monocytes, and type II alveolar epithelial cells [14]. Additionally, ficolin-1 concentrations in plasma are lower than those of ficolin-2 or ficolin-3 [21]. Our findings revealed that only ficolin-1 concentrations significantly differed between asthmatic patients and healthy controls.

In addition to recognizing pathogens, studies have revealed that ficolin-1 binds to natural killer (NK)‐cells and activates T-cell subsets through its FBG domain, creating a connection between innate and adaptive immunity [37]. Numerous studies have examined the involvement of ficolin-1 in the start and evolution of autoimmune and chronic inflammatory diseases. Hein et al. observed that the serum ficolin-1 in SLE patients was significantly reduced and linked to the severity of the condition in those with SLE [38]. Ammitzboll et al. and Kasperkiewicz et al. demonstrated that serum ficolin-1 levels were associated with increased disease activity in patients with rheumatoid arthritis [39] and juvenile idiopathic arthritis [40], respectively. Furthermore, both ficolin B deficiency and the administration of anti-ficolin-1 mAb were found to decrease the severity of collagen Ab–induced arthritis, with a reduction in the infiltration of synovial macrophages and neutrophils [41]. Elevated transcriptional levels of ficolin-1 in peripheral leukocytes and a heightened presence of ficolin-1-positive monocytes in glomeruli in individuals with microscopic polyangiitis have been documented [42].

Our data indicated a negative correlation between elevated ficolin-1 expression and pulmonary function, with asthma patients exhibiting worse lung function when their plasma ficolin-1 levels were high. This was further supported by a positive correlation between ficolin-1 concentrations and ACQ scores. These findings suggest that ficolin-1 may be a potential biomarker for clinical monitoring in asthma. Furthermore, our data showed that plasma ficolin-1 levels decreased after four weeks of ICS treatment. Taken together, these results suggest that elevated ficolin-1 may play a role in asthma development.

However, this study still has some limitations. First, though we found that asthma patients have higher levels of ficolin-1 in their plasma, we did not investigate the expression of ficolins in bronchoalveolar lavage fluid or induced sputum. Second, our study had a relatively small sample size, and may be subject to potential selection bias; however, our conclusion was considered acceptable, as there was no significant disparity between subjects with asthma and heathy controls regarding demographic factors. Thirdly, our study utilized ACQ questionnaire to score asthma symptoms, relied on self-reported data, and therefore may be subject to recall bias. Fourth, our study was a 4-week observational study, which was not sufficient to determine asthma severity, and therefore the association between asthma severity and ficolin-1 expression was not evaluated. Fifth, we only examined the expression of ficolin-1 in this study; further investigations will be conducted to explore the mechanism through in vitro studies and by utilizing a ficolin-1 knockout mouse model.

## Conclusions

Our research demonstrated the elevation of plasma ficolin-1 in asthma patients and correlations between its expression and lung function and ACQ score, thereby providing the first evidence of the effect of ficolin-1 on asthma. Collectively, ficolin-1 could be employed as a potential diagnostic marker and therapeutic target for asthma.

## Data Availability

The full data and materials of the current study are not publicly available due to limitations of ethical approval involving the patient data but are available from the corresponding author on reasonable request.

## Abbreviations

ICS: Inhaled corticosteroid
ACQ: Asthma control questionnaire
MBL: Mannose binding lectin
MASP: MBL-associated serine protease
ELISA: Enzyme-linked immunosorbent assay
FeNO: Fractional exhaled nitric oxide
IQR: Interquartile range
BMI: Body mass index
FEV_1_: Forced expired volume in 1 s
FVC: Forced vital capacity
ROC: Receiver operating characteristic;
AUC: Area under curve
CI: Confidence interval
SLE: Systemic lupus erythematosus
HC: Healthy control
